# Respiratory symptoms, exacerbations and sleep disturbances are more common among participants with asthma and chronic airflow limitation: an epidemiological study in Estonia, Iceland and Sweden

**DOI:** 10.1136/bmjresp-2023-002063

**Published:** 2024-02-19

**Authors:** Stephanie Mindus, Thorarinn Gislason, Bryndis Benediktsdottir, Rain Jogi, Robert Moverare, Andrei Malinovschi, Christer Janson

**Affiliations:** 1 Department of Medical Sciences: Respiratory, Allergy and Sleep Research, Uppsala University, Uppsala, Sweden; 2 Landspitali University Hospital, Reykjavik, Iceland; 3 Faculty of Medicine, University of Iceland, Reykjavik, Iceland; 4 Lung Clinic, Tartu, Estonia; 5 Thermo Fisher Scientific, Uppsala, Sweden; 6 Department of Medical Sciences: Clinical Physiology, Uppsala University, Uppsala, Sweden

**Keywords:** Asthma, COPD epidemiology, Asthma Epidemiology

## Abstract

**Background:**

Chronic airflow limitation (CAL) is a hallmark of chronic obstructive pulmonary disease but is also present in some patients with asthma. We investigated respiratory symptoms, sleep and health status of participants with and without CAL with particular emphasis on concurrent asthma using data from adult populations in Iceland, Estonia and Sweden investigated within the Burden of Obstructive Lung Disease study.

**Methods:**

All participants underwent spirometry with measurements of forced expiratory volume in 1 s (FEV_1_) and forced vital capacity (FVC) before and after bronchodilation. CAL was defined as postbronchodilator FEV_1_/FVC below the lower limit of normal. IgE-sensitisation and serum concentrations of eosinophil-derived neurotoxin (S-EDN) were assessed in a subsample. The participants were divided into four groups: no self-reported doctor’s diagnosed asthma or CAL, asthma without CAL, CAL without asthma and asthma and CAL: χ^2^ test and analysis of variance were used in bivariable analyses and logistic and linear regression when analysing the independent association between respiratory symptoms, exacerbations, sleep-related symptoms and health status towards CAL, adjusting for centre, age, sex, body mass index, smoking history and educational level.

**Results:**

Among the 1918 participants, 190 (9.9%) had asthma without CAL, 127 (6.6%) had CAL without asthma and 50 (2.6%) had CAL with asthma. Having asthma with CAL was associated with symptoms such as wheeze (adjusted OR (aOR) 6.53 (95% CI 3.53 to 12.1), exacerbations (aOR 12.8 (95% CI 6.97 to 23.6), difficulties initiating sleep (aOR 2.82 (95% CI 1.45 to 5.48), nocturnal gastro-oesophageal reflux (aOR 3.98 (95% CI 1.79 to 8.82)) as well as lower physical health status. In these analyses, those with no asthma and no CAL were the reference group. The prevalence of IgE-sensitisation was highest in both asthma groups, which also had higher levels of S-EDN.

**Conclusion:**

Individuals with self-reported asthma with CAL suffer from a higher burden of respiratory and sleep-related symptoms, higher exacerbation rates and lower health status when compared with participants with asthma alone or CAL alone.

**WHAT IS ALREADY KNOWN ON THIS TOPIC**

- Chronic airflow limitation (CAL) is a hallmark of chronic obstructive pulmonary disease but is also present in some individuals with asthma. In previous studies, CAL in asthma has been associated with a more severe disease and higher mortality compared with individuals with asthma without CAL.

**WHAT THIS STUDY ADDS**

- We find that individuals with asthma with CAL suffer from a higher burden of respiratory and sleep-related symptoms, high exacerbation rates and lower health status than participants with asthma alone or CAL alone.

**HOW THIS STUDY MIGHT AFFECT RESEARCH, PRACTICE OR POLICY**

- Participants with asthma with CAL need to be cared for differently or more intensely than other participants with respiratory symptoms and conditions. The management of this patient group should have a special focus on reducing exacerbation rates and improving sleep and health status.

## Introduction

Chronic obstructive pulmonary disease (COPD) and asthma are common respiratory diseases^1 2^ and overlap between the two are frequent.^3 4^ Persistent or chronic airflow limitation (CAL) is a hallmark of COPD^5^ and is mandatory for its diagnosis. Asthma is characterised by variable and reversible airflow limitation.^6^ However, some individuals with asthma also show chronic or fixed airflow limitation, attributed to airway remodelling.^7–9^ There is also evidence that loss of lung elasticity and even alveolar destruction, which result in mild alveolar dilation and reduced radial traction and mechanical support of airways, is an important mechanism causing CAL in asthma.^10^ Whereas a consensus definition of CAL is missing, airflow limitation is defined either as the ratio of forced expiratory volume in 1 s (FEV1) and forced vital capacity (FVC) being below a fixed value (70%)^11^ or below the lower limits of normal (LLN) (less than the fifth percentile) of a normally distributed set of values of FEV1/FVC for a population of non-smoking, normal individuals of the same age, height, sex and ethnicity.^12^

The mean prevalence of CAL in adults aged ≥40 years is 11.2% in men and 8.6% in women.^13^ Its prevalence in a non-smoking population is 5%.^14^ About 30%–50% of individuals with severe asthma have a CAL.^15^ Older age, longer duration of asthma and higher degree of severity of asthma are found in patients with CAL as compared with those without.^13 16^

Participants with asthma and CAL have an increased risk for elevated levels of sputum eosinophils (≥2%) and bronchial responsiveness,^13^ and CAL has been reported as a predictor of overall mortality in asthma.^17^ The aim of the present population-based study was, therefore, to investigate the clinical characteristics of participants with and without CAL with particular emphasis on concurrent asthma using data from adult populations in three Nordic Countries, all participating in the Burden of Obstructive Lung Disease (BOLD) study.

## Methods

The data are based on data from the BOLD study,^18^ an international collaboration to assess COPD’s prevalence and risk factors. Its design and rationale, the characteristics of its samples and the prevalence of chronic airflow obstruction, asthma and COPD have previously been published elsewhere.^18 19^ The BOLD study includes data on lung function, quality of life and exacerbations.^18 20^ The BOLD study in Iceland, Estonia and Sweden also included data on inflammatory markers^21 22^ and sleep-related variables.^23 24^

The participants were individuals aged 40 and over recruited through random sampling from the general population of Reykjavik, Iceland 2004–2005, Uppsala, Sweden 2006–2007 and Tartu, Estonia 2009. Information was obtained about demographics, respiratory symptoms and diagnoses, pharmacological treatment, smoking status (current, former, never), occupational exposure and education level through questionnaires in face-to-face interviews with trained and certified staff in the participant’s native language.

All participants underwent spirometry testing of FEV1 and FVC before and after bronchodilation (200 µg salbutamol). The methods developed for BOLD met or exceeded the American Thoracic Society standards for acceptable technique and equipment.^18 19 25^ The BOLD Pulmonary Function Reading Centre centrally reviewed all spirometry tests. The spirometry was conducted in the field, that is, not in a climate-controlled pulmonary function laboratory, with the participant sitting upright wearing a disposable mouthpiece and a nose clip. FEV1 and FVC values were obtained by spirometry using the ndd Easy OneTM Spirometer (ndd Medizintechnik, Zurich, Switzerland). Height and weight were measured, and body mass index (BMI) was calculated as weight in kilograms divided by the square of height expressed in metres.^18^ Chronic airflow obstruction (CAL) was defined as postbronchodilator FEV1/FVC below the lower LLN using the reference values from the third United States National Health and Nutrition Examination Survey for adult Caucasian men and women.^26^ The participants were divided into current, former and never-smokers. The participants were also asked about the highest level of schooling they had completed and categorised into three levels: elementary school, high school or university.

The following respiratory symptoms were analysed: wheeze—‘Have you had wheezing or whistling in your chest at any time during the last 12 months?’, wheeze only when having a cold—‘In the last 12 months, have you had this whistling in your chest only when you have a cold?’, wheeze in combination with breathlessness ‘In the last 12 months have you ever had an attack of wheezing or whistling that has made you feel short of breath?’, habitual cough—‘Do you usually cough when you don’t have a cold?’, and habitual phlegm—‘Do you usually bring up phlegm from your chest or do you usually have phlegm in your chest that is difficult to bring up when you don’t have a cold’. Asthma was defined as answering yes to the question: ‘Has a doctor or other health care provider ever told you that you have asthma, asthmatic bronchitis, or allergic bronchitis?’

Sleep-related symptoms were assessed using the Basic Nordic Sleep Questionnaire.^27^ The symptoms are assessed using a 5-point scale: 1, never or almost never; 2, less than once a week; 3, once or twice a week; 4, 3–5 nights/days a week and 5, almost every day or night. The following sleep-related symptoms were assessed: difficulties initiating sleep, excessive daytime sleepiness, snoring, nocturnal transpiration and witnessed apnoeas, where a report of having the symptom at least three nights/days per week was regarded as a positive response.^27^ Nocturnal gastro-oesophageal reflux (nGER) was also assessed. Having nGER-symptoms at least one night per week was regarded as a positive response.^28^ Difficulties maintaining sleep were also evaluated, where a response of every or almost every night was regarded as positive.

An exacerbation was defined as a period when the participant’s breathing got so bad that it interfered with usual daily activities or caused the participant to miss work during the last 12 months. We also asked whether the episode led to a healthcare contact or hospitalisation. Health status was assessed by the Short form (SF)-12 questionnaire (V.2),^29^ which is a generic instrument for assessing health status. The Physical Component Score (PCS) and Mental Health Component Score were calculated, with higher values indicating better health status.

Serum concentrations of interleukin 6 (IL-6) and C reactive protein (CRP) were for samples from all the centres measured at the University Hospital in Reykjavik as previously described.^21 22^ Serum IL-6 concentrations were measured with ELISA using reagents obtained from IBL (Hamburg, Germany). The lower detection limit of the IL-6 assay was 0.074 ng/L. CRP concentrations were measured on a Kone 30 analyser using a commercially available latex-enhanced immunoturbidimetric assay from Roche Diagnostic Systems (Mannheim, Germany). The lower detection limit of the assay was 0.1 mg/L. In the subsample from Uppsala, serum concentrations of eosinophil-derived neurotoxin (S-EDN) were expressed in ng/mL (Thermo Fisher Scientific Uppsala, Sweden). IgE sensitisation was assessed by measuring IgE antibodies in serum using ImmunoCAP Phadiatop (Phadia AB/Thermo Fisher Scientific, Uppsala, Sweden). The Phadiatop assay includes a mix of common perennial and seasonal aeroallergens, and the IgE antibody values are reported as Phadia arbitrary units per litre (PAU/L). Participants with IgE levels ≥0.35 PAU/L were regarded as atopic.^30^

All analyses were performed using Stata software, version intercooled STATA V.14.2 for Windows (Stata). In the analyses, the participants were divided into four groups: no asthma or CAL, asthma without CAL, CAL without asthma and asthma and CAL. In the bivariable analyses, the χ^2^ test was used to analyse categorical variables, while analysis of variance was used for continuous variables. Logistic regression was used when analysing the independent association between respiratory symptoms, exacerbations and sleep-related symptoms towards CAL, adjusting for centre, age, sex, BMI, smoking history (never, ex-smokers and current smokers) and educational level (elementary, secondary school, university). Multiple linear regression was used to analyse the association between health status and asthma and CAL groups. In the multivariable models, interaction for sex was also tested. Inflammatory markers levels below the detection value were replaced with a value that was half of that of the detection level (eg, CRP<0.1 mg/L was replaced with 0.05 mg/L). The inﬂammatory variables were not normally distributed and were log-transformed in the analyses and expressed as geometric mean with a 95% CI in the table. A p<0.05 denoted a statistically significant difference.

## Results

**Figure 1:**
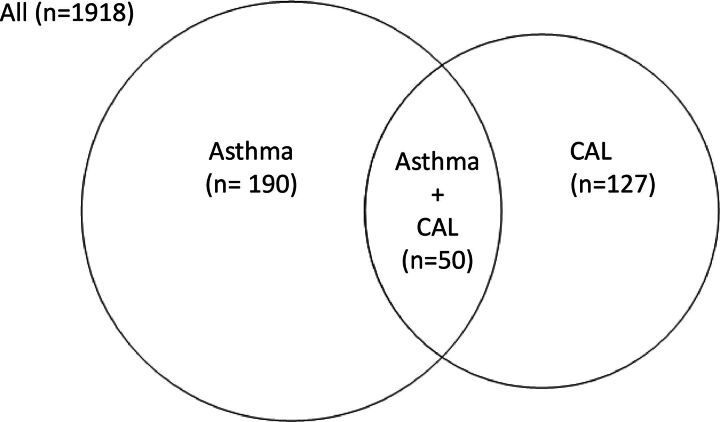
Distribution between participants with asthma alone, chronic airflow limitation (CAL) alone and asthma with CAL (asthma+CAL).

This analysis included 1918 participants, of which 190 (9.9%) belonged to the group asthma without CAL, 127 (6.6%) CAL without asthma and 50 (2.6%) CAL with asthma (figure 1). The characteristics of the four groups of participants are presented in table 1. Participants with CAL alone were more frequently men, ex-smokers or current smokers and were more likely to have a lower educational level. Participants with asthma with CAL shared some characteristics with the asthma group, such as female predominance and a low prevalence of current smoking, and others with the CAL groups, such as higher age, lower BMI and lower educational level. The highest use of asthma medication was found in the group with asthma with CAL.

**Table 1:** Characteristics of the participants (%, mean±SD) when divided on presence and absence of asthma, chronic airflow obstruction (CAL) and asthma with CAL

	Reference group (n=1551)	Only asthma (n=190)	Only CAL (n=127)	Asthma with CAL (n=50)	P value
Women	46.9	58.4	42.5	62.0	0.002
Age	57.8±11.5	58.0±11.4	63.7±11.9	65.3±12.8	<0.0001
BMI	27.9±6.0	29.3±6.1	26.4±4.8	26.4±3.7	0.0001
Smoke history					<0.0001
Never	46.2	44.7	24.4	38.0	
Former	37.4	42.1	44.9	44.0	
Current	16.4	13.2	30.7	18.0	
Educational level					<0.0001
Elementary	18.7	23.7	34.7	38.0	
High school	48.8	47.9	48.0	44.0	
University	32.6	28.4	17.3	18.0	
Medication*					
LABA	0.4	19.0	7.9	36.0	<0.0001
LAMA	0.1	1.0	4.7	2.0	<0.0001
ICS	2.1	45.3	9.4	60.0	<0.0001

The χ^2^ test was used in the statistical analyses.

*Any use in the last 12 months.

BMI, body mass index; ICS, inhaled corticosteroids; LABA, long-acting B2 agonist ; LAMA, long acting muscarinic antagonist.

**Figure 2:**
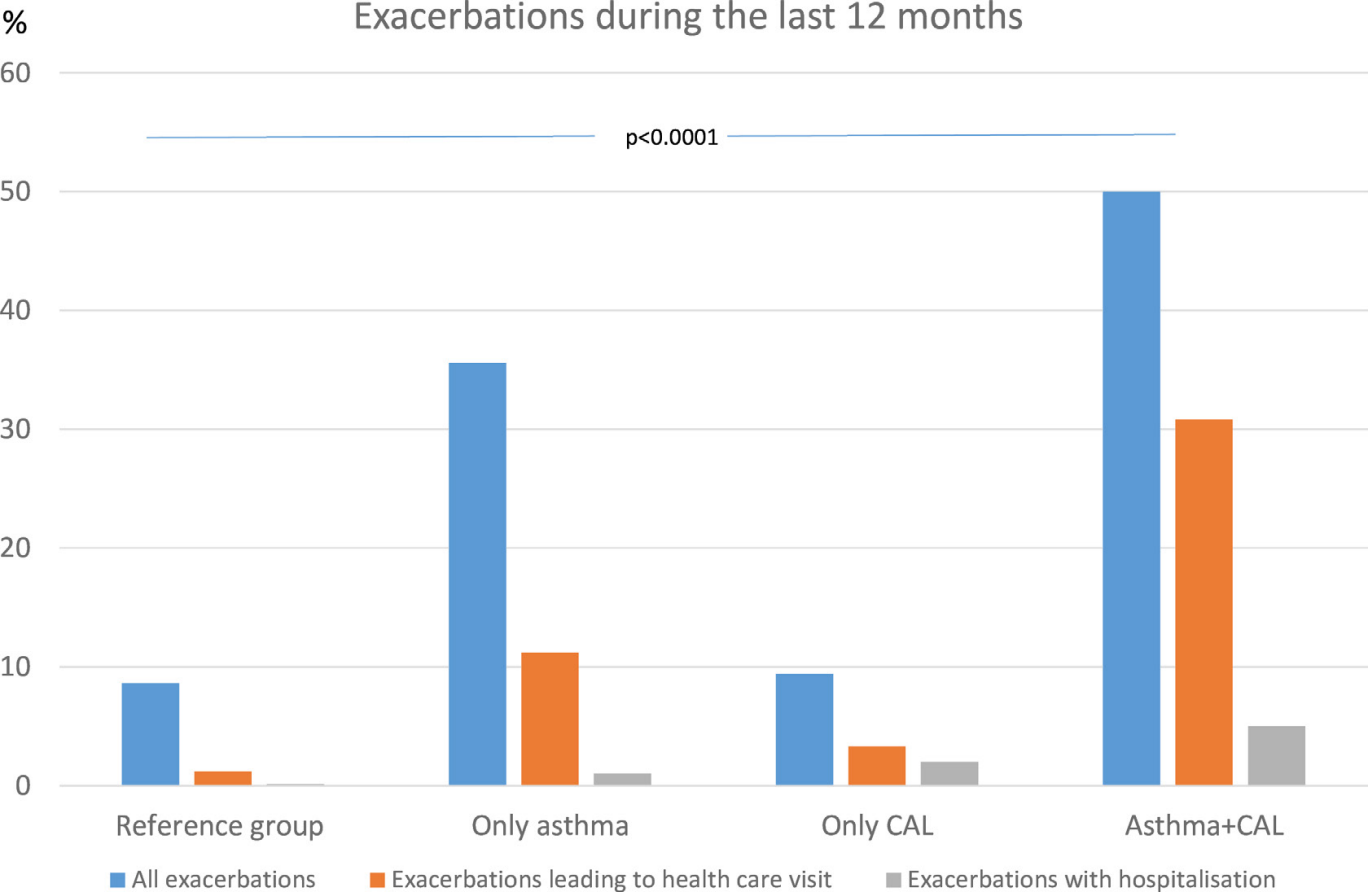
Prevalence of exacerbations in participants with asthma alone, chronic airflow limitation alone (CAL) and asthma with CAL (asthma+CAL). χ^2^ test was used in the statistical analyses.

The participants with asthma with CAL had the highest prevalence of respiratory symptoms and self-reported diagnosed chronic bronchitis and COPD (table 2). The group with asthma with CAL also had the highest prevalence of all kinds of exacerbations (figure 2). The prevalence of any exacerbation was 50% in the asthma with CAL group compared with 35% in the group with only asthma and 9% in those with only CAL and those without asthma and CAL. The corresponding figures for exacerbations leading to healthcare visits and exacerbation leading to hospitalisations were 31%, 11%, 3%, 1% and 5%, 1%, 2% and 0.1%, respectively. All group differences were highly significant (p<0.0001).

**Table 2:** The prevalence of respiratory and sleep-related symptoms (%, mean±SD) when divided on the presence and absence of asthma, chronic airflow obstruction (CAL) and asthma with CAL

	Reference group (n=1551)	Only asthma (n=190)	Only CAL (n=127)	Asthma with CAL (n=50)	P value
Wheeze	19.3	47.9	36.2	54.0	<0.0001
Wheeze only with a cold	10.1	16.8	16.5	18.0	0.003
Wheeze with breathlessness	4.3	24.2	7.1	28.0	<0.0001
Cough	20.1	45.8	37.0	48.0	<0.0001
Phlegm	16.3	35.3	28.4	50.0	<0.0001
Diagnosed Chronic bronchitis	2.4	14.2	6.3	42.0	<0.0001
Diagnosed COPD	0.3	3.2	8.7	20.0	<0.0001
Difficulties initiating sleep	15.0	19.1	20.0	36.4	0.001
Difficulties maintaining sleep	29.2	39.3	31.7	31.9	0.06
Excessive daytime sleepiness	21.7	32.8	24.3	29.6	0.01
Nocturnal gastro-oesophageal reflux	7.2	12.2	13.6	20.5	0.001
Snoring	29.3	29.9	34.7	29.7	0.81
Apnoeas	4.8	7.8	6.0	6.2	0.57
Nocturnal transpiration	12.6	24.6	14.3	20.4	0.42
MCS-12*	50.5±9.3	49.4±10.1	50.6±10.4	49.2±11.9	0.38
PCS-12†	48.5±9.3	45.7±11.1	44.7±11.2	40.1±13.1	<0.0001

The χ^2^ test was used for categorical variables and ANOVA for continuous variables.

*MCS-12: Mental Health Component Scale of the 12-item Short-Form Health Survey.

†PCS-12: Physical Component Summary Scale of the 12-item Short-Form Health Survey.

ANOVA, analysis of variance; COPD, chronic obstructive pulmonary disease.

Difficulties initiating sleep and nGER were most common among the group with asthma with CAL, while difficulties maintaining sleep and excessive daytime sleepiness were most often reported by those with asthma alone (table 2). The participants with asthma with CAL had the lowest mean physical component health status score of the groups, whereas no significant difference was found for the mental component score (table 2). No group differences were found for CRP and IL-6 (table 3). In the subsample of 498 participants from Uppsala, the prevalence of IgE-sensitisation was highest in the groups with asthma without CAL and asthma with CAL. These two groups also have higher levels of S-EDN (table 3).

**Table 3:** The prevalence of IgE sensitisation (%) and geometric mean (95% CI) of inflammatory markers when divided on the presence and absence of asthma, chronic airflow obstruction (CAL) and asthma with CAL

.	Reference group (n=1551)	Only asthma (n=190)	Only CAL (n=127)	Asthma with CAL (n=50)	P value
IgE sensitisation*(PAU/lL	21.4	58.6	24.2	40.0	<0.0001
CRP (mg/L)	1.5 (1.4 to 1.6)	1.7 (1.4 to 1.9)	1.4 (1.2 to 1.8)	1.4 (1.0 to 2.1)	0.56
IL-6 (ng/L)	1.2 (1.1 to 1.4)	1.4 (1.0 to 1.9)	1.7 (1.1 to 2.4)	1.2 (0.6 to 2.3)	0.17
S-EDN* ng/mL	18.6 (17.5 to 19.8)	24.6 (20.6 to 29.3)	21.1 (17.4 to 25.5)	26.4 (18.3 to 38.3)	0.005

ANOVA was used in the statistical analyses.

*Available from 498 participants, 397 in the reference group, 34 in asthma only, 33 CAL alone and 10 in the group with asthma and CAL.

ANOVA, analysis of variance; CRP, C reactive protein; IL-6, interleukin 6; ng/mL:, nanogram per millilitre; PAU/L, Phadia arbitrary units per litre; S-EDN, serum concentrations of eosinophil-derived neurotoxin.

**Figure 3:**
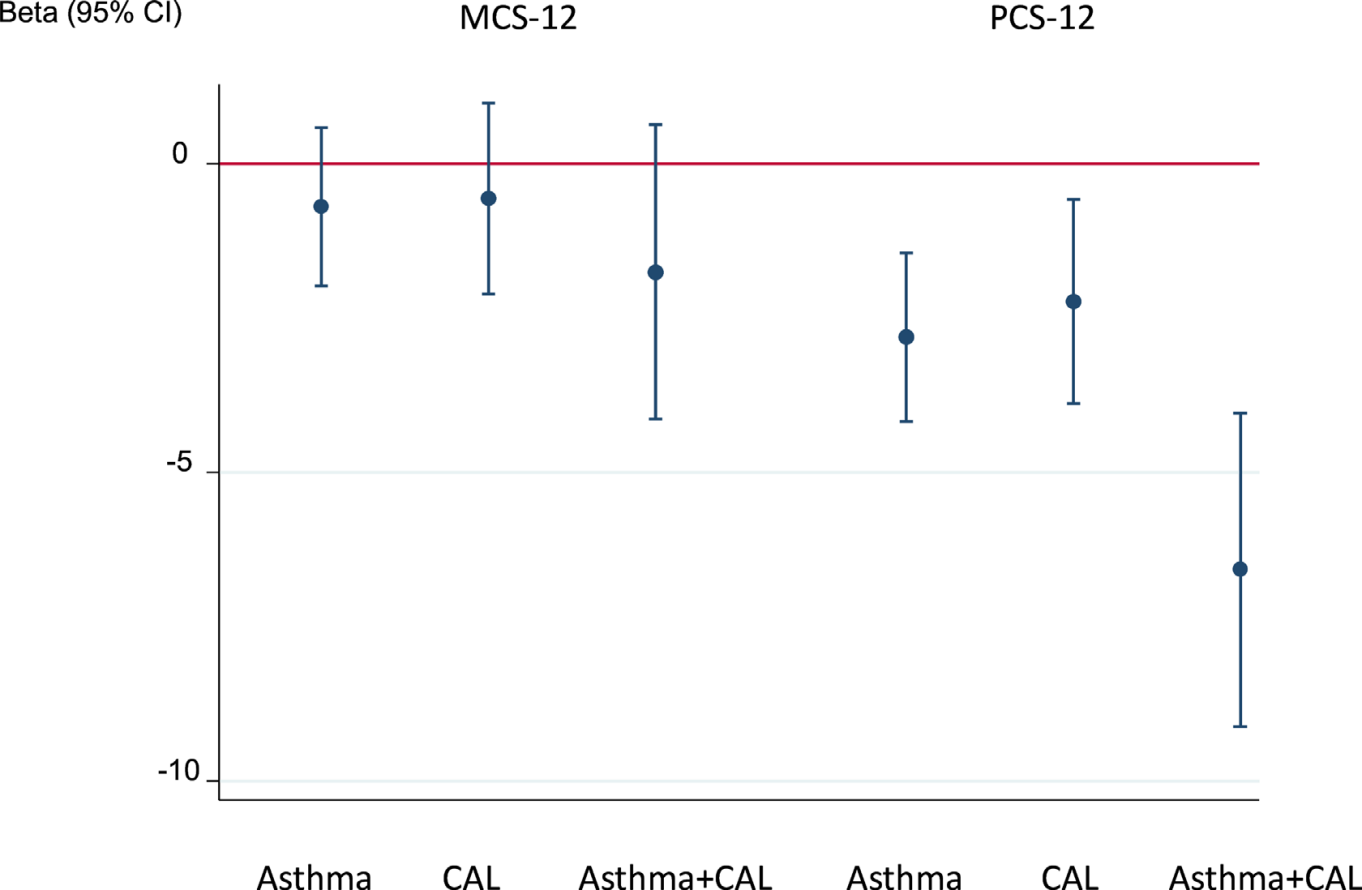
Independent association between health status and asthma, chronic airflow limitation (CAL) and asthma with CAL (adjusted* beta value with 95% CI). *Adjusted for sex, age, BMI, smoking history, educational level and centre. Multiple logistic regression was used in the statistical analyses. BMI, body mass index; MCS, Mental Component Score; PCS, Physical Component Score.

The strongest association with most respiratory symptoms, exacerbations, difficulty initiating sleep and nGER was seen in the group with asthma with CAL both in unadjusted (see online supplemental tables 1 and 2) and after adjustment for age, sex, BMI, smoking history, educational level and study centre using logistic regression (table 4). The same was true for the association to lower physical health status where the PCS was 5.5 (2.8 to 8.2) (beta (95% CI) lower in those with asthma and CAL than in those without asthma and CAL when analysed with multiple linear regression (figure 3). The strongest association with difficulties maintaining sleep and excessive daytime sleepiness was found in the group with asthma without CAL (table 4). There were no sex interactions regarding the association between CAL with asthma and symptoms, exacerbations or health status except that the association between nGER and asthma with CAL was stronger in men than women (OR (95% CI) 12.7 (3.63 to 44.2) vs 1.95 (0.64 to 5.95), p_interaction_=0.04) (see online supplemental tables 3 and 4).

10.1136/bmjresp-2023-002063.supp1 Supplementary data

**Table 4:** Independent association between respiratory and sleep-related symptoms and asthma, chronic airflow limitation (CAL) and Asthma with CAL

	Only asthma	Only CAL	Asthma with CAL
Wheeze	4.20 (2.98 to 5.92)	2.25 (1.48 to 3.43)	6.53 (3.53 to 12.1)
Wheeze only with a cold	1.82 (1.18 to 2.81)	1.52 (0.90 to 2.54)	1.95 (0.91 to 4.17)
Wheeze with breathlessness	7.11 (4.61 to 11.0)	1.49 (0.71 to 3.14)	8.58 (4.26 to 17.3)
Cough	3.44 (2.49 to 4.76)	1.87 (1.25 to 2.77)	3.24 (1.81 to 5.82)
Phlegm	3.45 (2.44 to 4.88)	1.58 (1.02 to 1.99)	5.01 (2.75 to 9.14)
Any exacerbation	6.24 (4.34 to 8.97)	1.28 (0.68 to 2.43)	12.8 (6.97 to 23.6)
Exacerbations with healthcare contact	9.81 (4.68 to 20.5)	3.33 (1.05 to 10.5)	48.1 (19.1 to 121)
Exacerbation with hospitalisation	18.8 (1.62 to 21.8)	37.0 (2.89 to 472)	110 (8.37 to 1439)
Difficulties initiating sleep	1.44 (0.94 to 2.22)	1.27 (0.75 to 2.16)	2.82 (1.45 to 5.48)
Difficulties maintaining sleep	1.76 (1.22 to 2.54)	0.88 (0.54 to 1.42)	0.86 (0.43 to 1.75)
Excessive daytime sleepiness	1.81 (1.27 to 2.59)	1.18 (0.73 to 1.92)	1.57 (0.80 to 3.09)
Nocturnal gastro-oesophageal reflux	2.10 (1.24 to 3.55)	2.33 (1.23 to 4.39)	3.98 (1.79 to 8.82)
Snoring	1.08 (0.70 to 1.67)	1.49 (0.82 to 2.38)	1.29 (0.60 to 2.76)
Apnoeas	1.66 (0.76 to 3.64)	1.37 (0.46 to 4.10)	1.90 (0.42 to 8.62)
Nocturnal transpiration	1.13 (0.71 to 1.81)	1.19 (0.66 to 2.15)	1.75 (0.81 to 3.79)

Multiple logistic regression was used in the statistical analyses.

The reference group comprised participants without CAL and asthma (adjusted OR* (95% CI)).

*Adjusted for sex, age, BMI, smoking history, educational level and centre.

BMI, body mass index.

## Discussion

In this study, based on large general population samples from Iceland, Estonia and Sweden, we observe that individuals with self-reported asthma with CAL are more often subject to exacerbations and have a larger burden of respiratory and sleep-related symptoms than those who had asthma without CAL or CAL without asthma.

We observe that participants with asthma with CAL were more often women than those with CAL alone or participants without CAL and asthma. Previous studies have also shown a female predominance in adults with asthma.^31^ Even so, the higher prevalence of adult women with asthma with CAL in the general population is a novel finding. Past studies have, in fact, shown asthma with CAL is more prevalent in adult men than women.^32–34^ We also observe that individuals with CAL alone are of male predominance, are of higher age, and have lower BMI and lower education, thus confirming previously published data.^19 35–37^ Furthermore, we observed a high prevalence of smoking among participants with CAL, with or without concurrent asthma. This result is also consistent with previous findings.^37^

The groups with asthma with CAL had the highest prevalence of all reported respiratory symptoms. The prevalence of exacerbations leading to a healthcare contact was almost tenfold that of what was observed among participants with CAL alone and nearly three times higher as compared with the asthma alone group. Participants with asthma with CAL are known to have higher exacerbation and hospitalisation rates and a higher burden of respiratory symptoms than their counterparts with asthma or CAL alone.^38^ The results are in line with what has been reported for participants with asthma COPD overlap (ACO), where this group also has been found to have more symptoms and a higher risk of exacerbations than those with asthma or COPD alone.^3 39–41^ This finding is perhaps not surprising since some studies on ACO have been defined in the same way as the asthma with CAL group in our study.^39^ Thus, it is plausible that the CAL with asthma and the CAL-alone groups included patients with CAL and synchronous asthma and COPD.

We also observe a high burden of sleep-related symptoms in individuals with asthma and CAL and in those with only asthma. Both asthma^42 43^ and COPD^44 45^ have previously been associated with sleep disturbances. Data on sleep quality in participants with CAL and asthma are still scarce. We have previously shown that participants with asthma and COPD overlap (ACO) suffer from a higher burden of insomnia symptoms, that is, difficulty initiating sleep, difficulty maintaining sleep, early morning awakenings and excessive daytime sleepiness.^3^ Our previous study was, however, mostly questionnaire based, whereas in this study, all participants had done spirometry. Another advantage of this study is that we had health status data. In the study, participants with both asthma with CAL showed a greater deterioration in their physical health status than their counterparts with CAL or asthma alone. However, no significant difference between groups was found regarding mental health status. This finding is in line with previous studies reporting that participants with ACO have a lower quality of life than those with asthma or COPD alone.^39 40^ It is possible that the decreased health status in the asthma and CAL groups is at least to some extent explained by the increased prevalence of sleep disturbances.

The two groups of patients with asthma had the highest levels of S-EDN and the highest prevalence of IgE-sensitisation. Asthma encompasses different endotypes, characterised by different inflammatory pathways and responsiveness to therapies. There are two main inflammatory endotypes: T2-high and T2-low.^46^ Increased levels of T2 inflammation biomarkers such as the fraction exhaled nitric oxide (FENO), S-EDN and urinary-EDN and serum eosinophil cationic protein (S-ECP) have been linked to decreased lung function markers^47–49^ and may actively injure the lungs and thus, partake in the remodelling process^50^ leading to CAL. Simultaneously elevated nitric oxide and serum-eosinophil cationic protein have also been linked to asthma exacerbations.^50^ Some biomarkers such as immunoglobulin E (IgE), sputum or peripheral blood eosinophil count and FENO have also been suggested as helpful when choosing treatment and assessing the prognosis of COPD,^51^ identifying COPD patients with possible synchronous asthma. Still, they are not diagnostic for asthma with CAL.^52 53^

We found no group differences for markers of systemic inflammation (CRP and IL-6). CRP is an acute-phase protein frequently used as a surrogate marker for inflammation. IL-6 is a proinflammatory cytokine. Both have been commonly studied in asthma and COPD.^21 54^ Previous data have shown that systemic inflammation can be related to both COPD^55^ and asthma^56^ and low levels of FEV1 and FVC rather than to their ratio.^21 57^

The inherent strength of our work is that it is based on large population samples from three different countries and that validated questionnaire tools have been used. We add further evidence to the discussion, as we have both type 2 and systemic inflammation markers at our disposal. We have also chosen the LLN for the definition of CAL rather than using the fixed ratio definition with postbronchodilation FEV1/FVC, thus reducing the number of misclassified spirometries. We are aware of the limitations of our study. Our data’s cross-sectional nature may limit the validity of our conclusions. Moreover, the asthma diagnosis was self-reported, and, as in all questionnaire-based studies, selection and recall bias may be present.

In conclusion, we observe that individuals with the combination of asthma and CAL suffer from a higher burden of respiratory and sleep-related symptoms, including higher exacerbation rates, difficulties initiating sleep, nGER and lower disease-related quality of life than participants with asthma alone or CAL alone. These results indicate that participants with asthma with CAL need to be cared for differently or more intensely than other participants with respiratory symptoms and conditions.

## Data Availability

Data are available on reasonable request. The dataset is still subject to further analyses but will continue to be held and managed by the Department of Medical Sciences, Uppsala University, Uppsala, Sweden. Relevant anonymised data are available on reasonable request from the authors.
