# Supplementary material for: Respiratory symptoms, exacerbations and sleep disturbances are more common among participants with asthma and chronic airflow limitation: an epidemiological study in Estonia, Iceland and Sweden
Source: BMJ Open Respir Res. 2024 Feb 19;11(1):e002063. doi: 10.1136/bmjresp-2023-002063 (PMC10882325; doi:10.1136/bmjresp-2023-002063)
Supplement: Supplementary data [file bmjresp-2023-002063supp001.pdf]

Online Table 1. Association between respiratory and sleep-related symptoms and asthma, chronic airflow limitation (CAL) and Asthma with CAL. The reference group comprised subjects without CAL and asthma (Odds ratio* (95% confidence interval).

	Only asthma	Only CAL	Asthma with CAL
Wheeze	3.85 (2.82-5.25)	2.38 (1.62-3.49)	4.92 (2.78-8.69)
Wheeze without cold	1.81 (1.20-2.74)	1.77 (1.08-2.91)	1.96 (0.94-4.11)
Wheeze with breathlessness	7.07 (4.68-10.7)	1.69 (0.82-3.47)	8.60 (4.43-16.7)
Cough	3.35 (2.46-4.58)	2.33 (1.59-3.41)	3.66 (2.07-6.47)
Phlegm	2.79 (2.02-3.87)	2.03 (1.35-3.05)	5.13 (2.90-9.08)
Any exacerbation	5.81 (4.11-8.21)	1.11 (0.60-2.07)	10.7 (5.96-19.1)
Exacerbations with health care contact	10.7 (5.28 -21.7)	2.93 (0.97-8.84)	37.7 (16.4-86.6)
Exacerbation with hospitalisation	20.7 (1.86-2.29)	24.7 (2.22-275)	78.9 (7.00-889)
Difficulties initiating sleep	1.33 (0.89-2.01)	1.41 (0.86-2.34)	3.23 (1.72-6.09)
Difficulties maintaining sleep	1.57 (1.13-2.18)	1.12 (0.73-1.73)	1.13 (0.59-2.15)
Excessive daytime sleepiness	1.76 (1.24-2.49)	1.16 (0.72-1.85)	1.51 (0.782.93)
Nocturnal gastroesophageal reflux	1.80 (1.08-2.97)	2.03 (1.11-3.71)	3.32 (1.55-7.12)
Snoring	1.03 (0.69-1.54)	1.28 (0.78-2.10)	1.02 (0.50.2.09)
Apneas	1.68 (0.80-3-55)	1.26 (0.44-3.62)	1.32 (0.31-5.71)
Nocturnal transpiration	1.19 (0.75-1.88)	1.16 (0.65-2.05)	1.79 (0.84-3.79)

Simple logistic regression was used in the analyses.

Online table 2. Independent association between health status asthma with CAL (Beta value with 95% confidence interval) in men and women (MCS= mental component score, PCS = physical component score).

	Only asthma	Only CAL	Asthma with CAL
MCS	-1.09 (-2.53, 0.34)	0.13 (-1.59, 1.86)	-1.29 (-3.97, 1.40)
PCS	-2.75 (-4.22, -1.29)	-3.74 (-5.50, -1.98)	-8.40 (-11.1, -5.65)

Simple linear regression was used in the analyses.

Online table 3. Independent association between respiratory and sleep-related symptoms Asthma with CAL in men and women. The reference group comprised subjects without CAL and asthma (Adjusted Odds ratio* (95% confidence interval).

	Men<br>Asthma with CAL	Women<br>Asthma with CAL	$p_{interaction}$
Wheeze	3.42 (1.31-8.90)	12.0 (5.31-27.0)	0.07
Wheeze without cold	0.87 (0.19-3.91)	3.38 (1.34-8.53)	0.12
Wheeze with breathlessness	7.79 (2.25-27.0)	10.2 (4.25-24.6)	0.46
Cough	3.39 (1.31-8.80)	3.06 (1.44-6.49)	0.96
Phlegm	2.65 (0.99-7.09)	7.37 (3.39-16.0)	0.10
Any exacerbation	7.07 (2.48-20.2)	17.4 (7.83-38.8)	0.08
Exacerbations with healthcare contact	49.0 (7.09-338)	46.3 (15.5-138)	0.80
Difficulties initiating sleep	1.34 (0.28-6.45)	3.65 (1.68-7.92)	0.23
Difficulties maintaining sleep	0.43(0.11-1.74)	1.23 (0.53-2.84)	0.22
Excessive daytime sleepiness	1.63 (0.48-5.52)	1.53 (0.68-3.45)	0.98
Nocturnal gastroesophageal reflux	12.7 (3.63-44.2)	1.95 (0.64-5.95)	0.04
Snoring	1.70 (0.48-5.96)	1.20 (0.44-3.26)	0.78
Apneas	1.71 (0.19-15.1)	2.45 (0.28-21.3)	0.83
Nocturnal transpiration	2.36 (0.49-11.4)	1.74 (0.71-4.24)	0.99

*Adjusted for age, BMI, smoking history, educational level and centre.

Multiple logistic regression was used in the analyses

Online table 4. Independent association between health status asthma with CAL (adjusted* Beta value with 95% confidence interval) in men and women (MCS= mental component score, PCS = physical component score).

	Men<br><br>Asthma with CAL	Women<br><br>Asthma with CAL	$p_{interaction}$
MCS	-1.69 (-5.09, 1.71)	-1.96 (-5.33, 1.40)	0.95
PCS	-8.50 (-12.3, -4.75)	-5.70 (-9.19, -2.21)	0.42

*Adjusted for sex, age, BMI, smoking in men and history, educational level and centre.

Multiple linear regression was used in the analyses
